# Impact of immunohistochemical expression of kinesin family member 18A (Kif18A) and β-catenin in infiltrating breast carcinoma of no special type

**DOI:** 10.1186/s12957-023-03276-3

**Published:** 2024-01-10

**Authors:** Aiat Shaban Hemida, Mohammed Ibrahim Shabaan, Mennatallah Ahmed Taha, Asmaa Gaber Abdou

**Affiliations:** Pathology Department, Faculty of Medicine, Menoufia University, Yassin Abd Elghafar Street, Shebin El Kom, 32511 Egypt

**Keywords:** KIF18A, β-catenin, Breast carcinoma

## Abstract

**Background:**

KIF18A is a regulator of the cell cycle that stimulates the proliferation of cancer cells. The Wnt/β-catenin pathway is involved in different issues’ carcinogenesis and is being examined as a therapeutic target. The relationship between KIF18A and β-catenin in breast cancer was not previously investigated. Therefore, this work aims to study the immunohistochemical expression and correlation of KIF18A and β-catenin in breast-infiltrating duct carcinoma (IDC) and their relation to prognosis.

**Material and methods:**

Slides cut from paraffin blocks of 135 IDC and 40 normal breast tissues were stained by KIF18A and β-catenin antibodies. KIF18A cytoplasmic or nucleocytoplasmic staining and β-catenin aberrant expression either nucleo-cytoplasmic or cytoplasmic staining were considered.

**Results:**

Normal breast tissue and IDC showed a significant difference regarding KIF18A and aberrant β-catenin expression. High KIF18A and β-catenin *H* score values were associated with poor prognostic factors such as high grade, advanced stage, distant metastasis, high Ki67 status, and Her2neu-enriched subtype. There was a significant direct correlation between KIF18A and β-catenin as regards percent and H score values. Prolonged overall survival (OS) was significantly associated with mild intensity and low *H* score of KIF18A, and low β-catenin *H* score.

**Conclusions:**

KIF18A could be involved in breast carcinogenesis by activating β-catenin. Overexpression of KIF18A and aberrant expression of β-catenin are considered proto-oncogenes of breast cancer development. KIF18A and β-catenin could be poor prognostic markers and predictors of aggressive behavior of breast cancer.

## Introduction

Currently, breast cancer is one of the most prevalent cancers according to the GLOBOCAN 2020 data. It is the 5th cause of cancer-related deaths, and about 2.3 million new cases are diagnosed worldwide [1]. Breast cancer incidence seems to be slightly lower in Egypt than in the USA and other Western countries. However, Egyptian breast cancer patients are characterized by a higher mortality rate than the USA [2].

Screening, diagnosis, and early management became a goal to increase the survival of breast cancer patients. However, recurrence, metastasis, and resistance to treatment persist [3, 4]. A lot of molecules are shared in breast cancer pathogenesis, including neuropeptide substance P and its receptor (NK1R) and other molecules [5]. Therefore, it became mandatory to search for molecular mechanisms and prognostic biomarkers for the prediction of breast cancer behavior.

The kinesin protein superfamily includes the KIF18A protein, which acts as intracellular motor transporters through microtubules. KIFs have important roles in different biological pathways for example cellular morphogenesis and mitosis pathways. They also participate in the pathogenesis of different tumors [6].

KIF18A is a regulator of the cell cycle acting on the G2/M phase, and if it is knocked down, the proliferation of cancer cells is inhibited [7]. In breast cancer, few previous studies investigated the significance of KIF18A expression [8–10], which are not enough to establish its potential role in breast cancer.

Regulation of Wnt/β-catenin activity is associated with carcinogenesis in different tissues and is being investigated as therapeutic targets [11]. In cervical cancer, it was found that KIF18B acts as an oncogene that promotes cervical carcinogenesis by activating the Wnt/β-catenin signaling pathway [12]. However, to the best of our knowledge, the relationship between KIF18A and β-catenin in breast cancer was not previously investigated. Therefore, this work aims to study the immunohistochemical expression and correlation of KIF18A and β-catenin in breast infiltrating duct carcinoma (IDC) and to find out their possible prognostic significance.

## Material and methods

This is a retrospective study that was done on archival material from 135 IDC breast and 40 control group paraffin blocks. They were obtained from the Pathology Department, Faculty of Medicine, Menoufia University Hospital, between January 2018 and January 2021. The protocol of this work was approved by the institutional ethical committee (IRB: 10/2020 PATH 39).

The studied cases were categorized into 40 control group cases (normal breast tissue from reduction mammoplasty done at the plastic surgery department) and 135 IDC breast cases (modified radical mastectomy was the procedure in 115 cases while 20 cases underwent conservative breast surgery). Cases subjected to neo-adjuvant therapy and histopathologic types other than IDC were excluded from the study.

### Clinicopathological data

Clinical parameters were collected from patients’ medical records. Data concerning histopathologic features were obtained by revision of pathology reports and re-evaluation of hematoxylin and eosin (H&E) stained sections. Staging is based on the TNM staging system and according to the updated (8th) edition of the American Joint Committee on Cancer (AJCC) [13]. They were grouped into two categories; early (T1 and T2) and advanced (T3 and T4). Grading was performed according to Elston and Ellis, 1991 [14]. Assessment of Nottingham prognostic index (NPI) [15], stromal tumor-infiltrating lymphocytes (TILs) within the borders of the invasive tumor [16], and lympho-vascular and perineural invasion were done [17, 18]. Ductal carcinoma in situ (DCIS) was evaluated regarding its presence, extent, and grade. Mitoses were counted [19]. Molecular classification was done based on ER, PR, Her2neu, and Ki-67 immunostaining [20].

### Tissue microarray (TMA) blocks

Duplicate-core TMA blocks were processed using a manual tissue microarrayer (Breecher instrument Manual Microarray, Wisconsin, USA).

### Immunohistochemistry

The technique used is the streptavidin-biotin amplified immunostaining system. The primary antibodies were incubated overnight. KIF18A rabbit polyclonal antibody [concentrated form (100 ul) with a dilution of 1:50, (Chongqing Biospes), Catalog # YPA2293] and β-catenin rabbit polyclonal antibody [concentrated form (100 ul) with a dilution of 1:100, (Chongqing Biospes), Catalog # YPA1606] were used. The sections were treated with tris-EDTA high PH retrieval solution (Dako, Ref K8000, Glostrup, Denmark). The positive control was a normal human colonic tissue for KIF18A and fibromatosis for B-catenin. Sections stained with the omission of the primary antibodies step were included as a negative control.

### Immunohistochemical staining interpretation

Regarding KIF18A, cytoplasmic or nucleocytoplasmic staining was considered positive in any number of cells. β-catenin positive stain was defined when membranous staining of cells; aberrant expression was nucleo-cytoplasmic or cytoplasmic staining in >5% tumor cells [19].

For each immunohistochemical marker, staining intensity was assessed, either 0 (negative), 1 (weak), 2 (medium), or 3 (strong). The percentage of staining was estimated. *H* score was calculated according to the following equation [21]:

$$H-\;\mathrm{score}=1\;\times\;\%\;\mathrm{of}\;\mathrm m\mathrm i\mathrm l\mathrm d\mathrm l\mathrm y\;\mathrm s\mathrm t\mathrm a\mathrm i\mathrm n\mathrm e\mathrm d\;\mathrm c\mathrm e\mathrm l\mathrm l\mathrm s\;+\;2\;\times\;\%\;\mathrm m\mathrm o\mathrm d\mathrm e\mathrm r\mathrm a\mathrm t\mathrm e\mathrm l\mathrm y\;\mathrm s\mathrm t\mathrm a\mathrm i\mathrm n\mathrm e\mathrm d\;\mathrm c\mathrm e\mathrm l\mathrm l\mathrm s\;+\;3\;\times\;\%\;\mathrm o\mathrm f\;\mathrm s\mathrm t\mathrm r\mathrm o\mathrm n\mathrm g\mathrm l\mathrm y\;\mathrm s\mathrm t\mathrm a\mathrm i\mathrm n\mathrm e\mathrm d\;\mathrm c\mathrm e\mathrm l\mathrm l\mathrm s$$

Cases were then classified as low and high *H* score groups according to the median value of *H* score.

### Survival data analysis

Data was collected from patient’s files at the Clinical Oncology and Nuclear Medicine Department, Faculty of Medicine, Menoufia University. The overall survival (OS) was calculated from the date of primary breast cancer diagnosis to the date of death or last follow-up. The follow-up period extended from January 2018 to December 2022. The survival time ranged from 9 to 52 months with a mean± SD of 28.24±13.52 months and a median of 30 months. Kaplan-Meier survival curves and Hazard function curves were constructed for survival analysis [22].

### Statistical analysis

Statistical Package for Social Science (SPSS) version 22 (SPSS Inc., Chicago, USA) was used. Qualitative data were expressed in numbers and percentages. Quantitative data were described using range, interquartile range (IQR), mean (*χ*^2^), standard deviation (SD), and median. Chi‐square (*χ*^2^ test), Fisher’s exacts, Student’s t, ANOVA, Mann-Whitney *U* (*U* test), Kruskal–Wallis, McNemar, and marginal homogeneity tests were used. Pearson’s correlation coefficient is a measure of the linear correlation between two continuous variables. Differences were considered statistically significant when *P* value was ≤0.05. *P* value <0.01 was statistically highly significant [23].

## Results

### Clinical and pathological data of IDC cases

The clinical and pathological data of IDC cases are presented in Table 1.

**Table 1:** Clinicopathological data of the studied breast cancer cases (*n* = 135)

**Variable**		**No.**	**%**
**Age**	Min. – Max.	30.0–82.0	
	Mean ± SD.	55.39 ± 11.73	
	Median (IQR)	55.0 (48.0–65.0)	
**Size**	Min. – Max.	0.50–15.0	
	Mean ± SD.	3.63 ± 2.20	
	Median (IQR)	3.0 (2.0–5.0)	
**T stage grouping**	Early	105	77.8
	Advanced	30	22.2
**Nodal metastasis**	No	31	23.0
	Yes	104	77.0
**AJCC stage grouping**	Early	63	46.7
	Advanced	72	53.3
**Distant metastasis**	No	121	89.6
	Yes	14	10.4
**Grade**	Grade I	2	1.5
	Grade II	113	83.7
	Grade III	20	14.8
**Grade**	Low grade	115	85.2
	High grade	20	14.8
**Necrosis**	Absent	97	71.9
	Present	38	28.1
**Mitosis**	Min. – Max.	1.0–23.0	
	Mean ± SD.	3.90 ± 5.24	
	Median (IQR)	1.0 (1.0–3.0)	
**NPI score**	Min. – Max.	2.40–8.0	
	Mean ± SD.	5.11 ± 1.28	
	Median (IQR)	5.20 (4.20–6.0)	
**NPI group**	Poor	59	43.7
	Moderate	55	40.7
	Good	21	15.6
**Tumor infiltrating lymphocytes**	Absent	48	35.6
	Present	87	64.4
**Lymphovascular invasion**	No	127	94.1
	Yes	8	5.9
**Grade of DCIS (no=74)**	Low grade	21	28.4
	High grade	53	71.6
**ER**	Negative	29	21.5
	Positive	106	78.5
**PR**	Negative	41	30.4
	Positive	94	69.6
**Her2neu**	Negative	84	62.2
	Positive	51	37.8
**Ki 67 proliferative index**	Low	79	58.5
	High	56	41.5
**Molecular subtype**	Luminal	106	78.5
	Her2neu-enriched	18	13.3
	Triple negative	11	8.2

*IQR* interquartile range, *SD* standard deviation, *NPI* Nottingham prognostic index, *DCIS* ductal carcinoma in situ

### Expression of KIF18A in studied groups (IDC and control normal breast tissue groups)

**Fig. 1:**
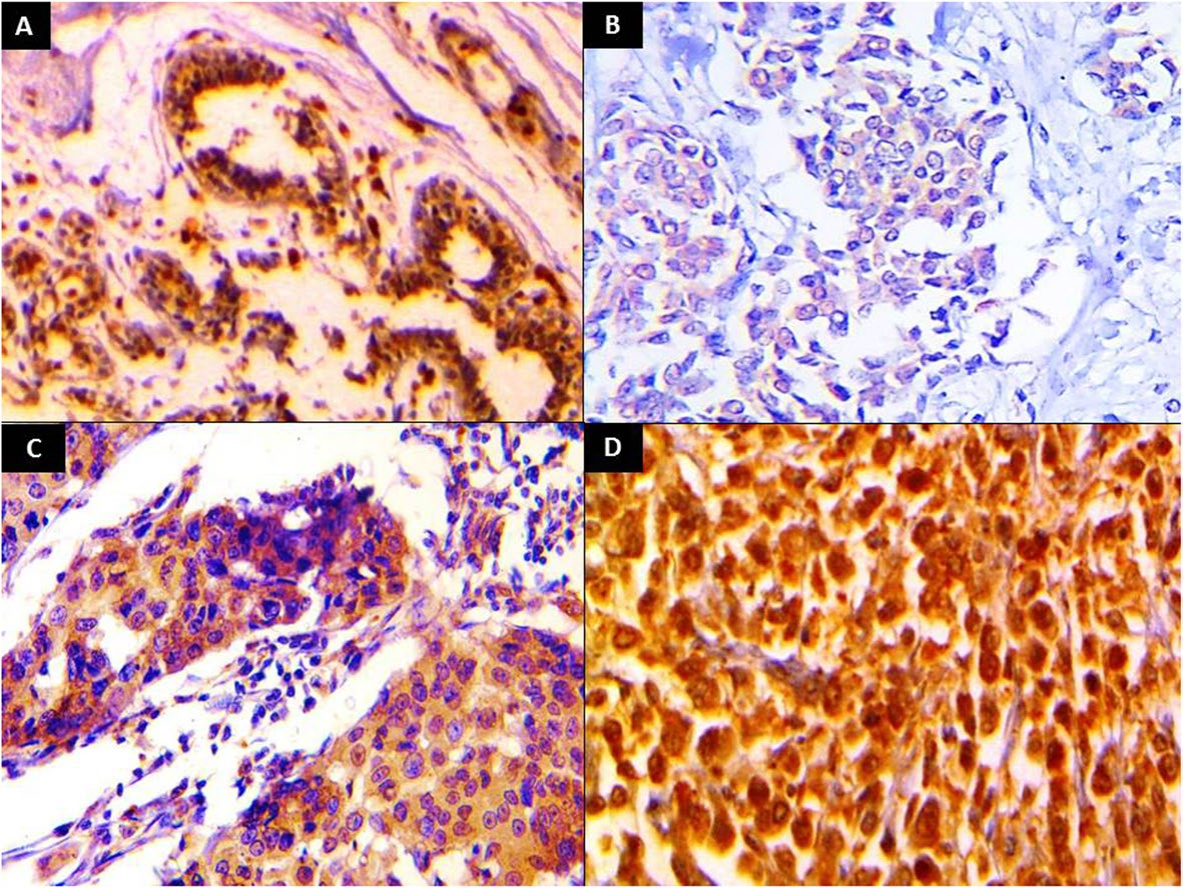
KIF18A immunostaining showed **A** low expression in normal breast tissue (IHC x40). Infiltrating duct carcinoma showed **B** mild nucleocytoplasmic expression of KIF18A (IHC x100), **C** moderate cytoplasmic expression of KIF18A (IHC x200), and **D** strong nucleocytoplasmic expression of KIF18A (IHC x200)

All control group specimens showed positive nucleocytoplasmic expression of KIF18A protein (100%), and KIF18A *H* score ranged from 65 to 180 with a mean± SD of 129±35.41 and a median of 140 (Fig. 1A).

One hundred thirty-four (99.3%) IDC cases showed positive expression of KIF18A. Seventy-five cases (56%) showed a nucleo-cytoplasmic pattern of KIF18A expression, while 59 cases (44%) showed a cytoplasmic pattern of expression. KIF18A *H* score ranged from 0 to 285 with a mean ±SD of 197.93 ± 64.80 and a median of 210 (Fig. 1B, C, D).

### Comparison between IDC and control normal breast tissue groups as regards KIF18A expression

There was a highly significant difference between IDC and control normal breast tissue groups regarding KIF18A percent, intensity, and mean *H* score expression (*p*<0.001 for all) (Table 2).

**Table 2:** Comparison between the studied groups (IDC and control normal breast tissue groups) regarding KIF18A immunostaining

**KIF18A**		**Cases (*n* = 135)**		**Control normal breast (*n* = 40)**		**Test of sig.**	***P***
		**No.**	**%**	**No.**	**%**		
**Positivity**	Negative	1	0.7	0	0.0	*χ*^2^=0.298	^MC^*p*=1.000
	Positive	134	99.3	40	100.0		
**Localization**	Cytoplasmic	59	44.0	0	0.0	*χ*^2^=119.814^*^	<0.001^*^
	Nucleocytoplasmic	75	56.0	39	100		
**Percent**	Min.–Max.	0.0–95.0		40.0–80.0		*U*=1260.50^*^	<0.001^*^
	Mean ± SD.	74.78 ± 14.99		63.0 ± 11.70			
	Median (IQR)	80.0 (70.0–85.0)		62.50(52.50–75.0)			
**Intensity**	Mild	9	6.7	9	22.5	*χ*^2^=17.851^*^	<0.001^*^
	Moderate	34	25.4	18	45.0		
	Strong	91	67.9	13	32.5		
	Mild + moderate	43	32.1	27	67.5	*χ*^2^=16.064^*^	<0.001^*^
	Strong	91	67.9	13	32.5		
***H* score**	Low (<210)	59	43.7	40	100.0	*χ*^2^=39.085^*^	<0.001^*^
	High (>210)	76	56.3	0	0.0		
	Min.–Max.	0.0–285.0		65.0–180.0		*U*=953.50^*^	<0.001^*^
	Mean ± SD.	197.93 ± 64.80		129.0 ± 35.41			
	Median (IQR)	210 (160.0–255.0)		140 (100.0–150.0)			

*IQR* interquartile range, *SD* standard deviation, *U* Mann-Whitney test, *χ*^*2*^ chi-square test, *MC* Monte Carlo, *p p* value for comparing between the studied groups

^*^Statistically significant at *p* ≤ 0.05

#### Relationship between KIF18A expression and clinicopathological data in IDC cases (*n* = 135)

**Fig. 2:**
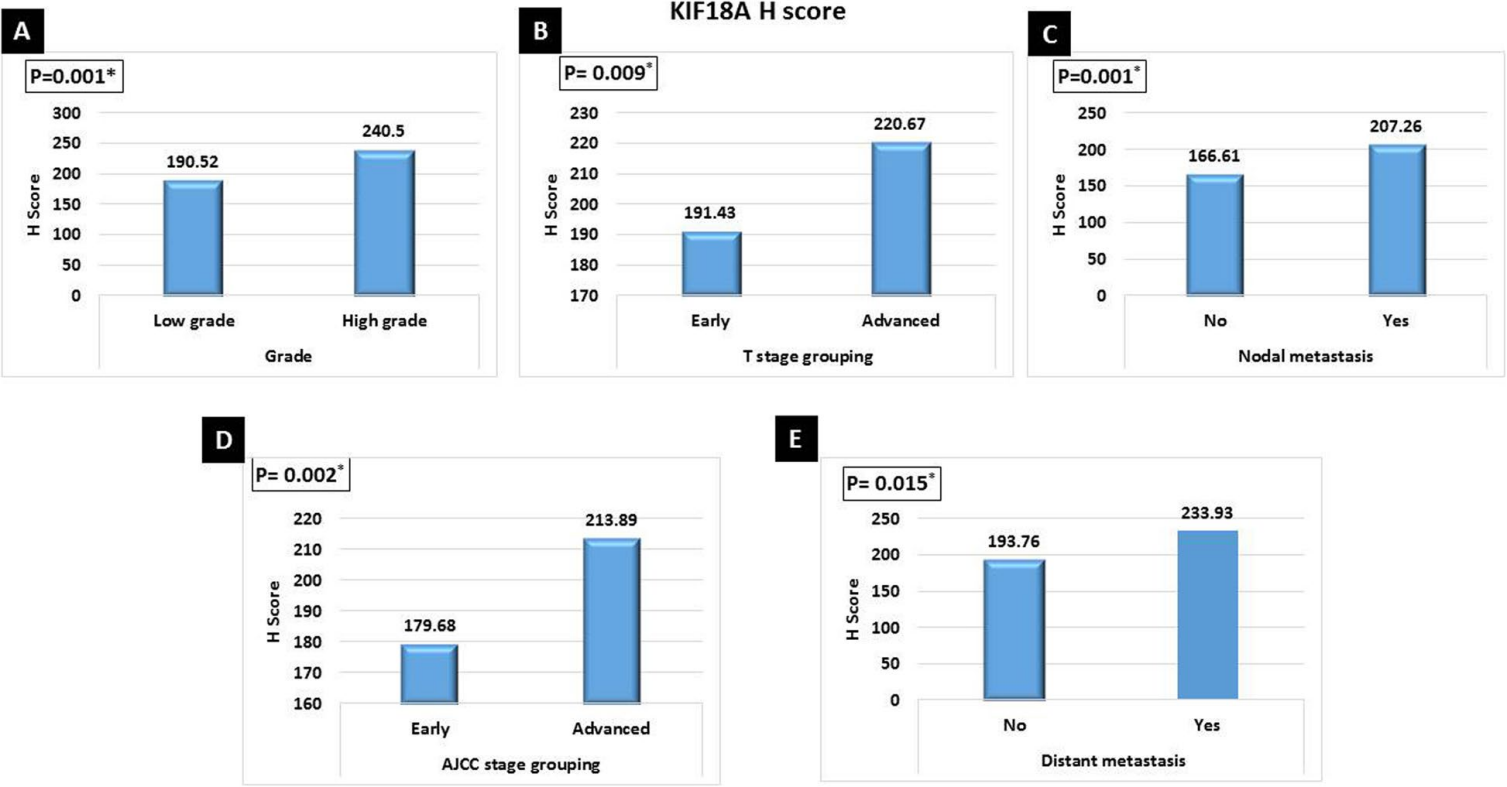
Significant relation between high KIF18A *H* score value expression and **A** high tumor grade (*p*=0.001), **B** advanced tumor stage grouping (*p*=0.009), **C** nodal metastasis (*p*=0.001), **D** advanced AJCC stage grouping (*p*=0.002), and **E** distant metastasis (*p*=0.015)

**Fig. 3:**
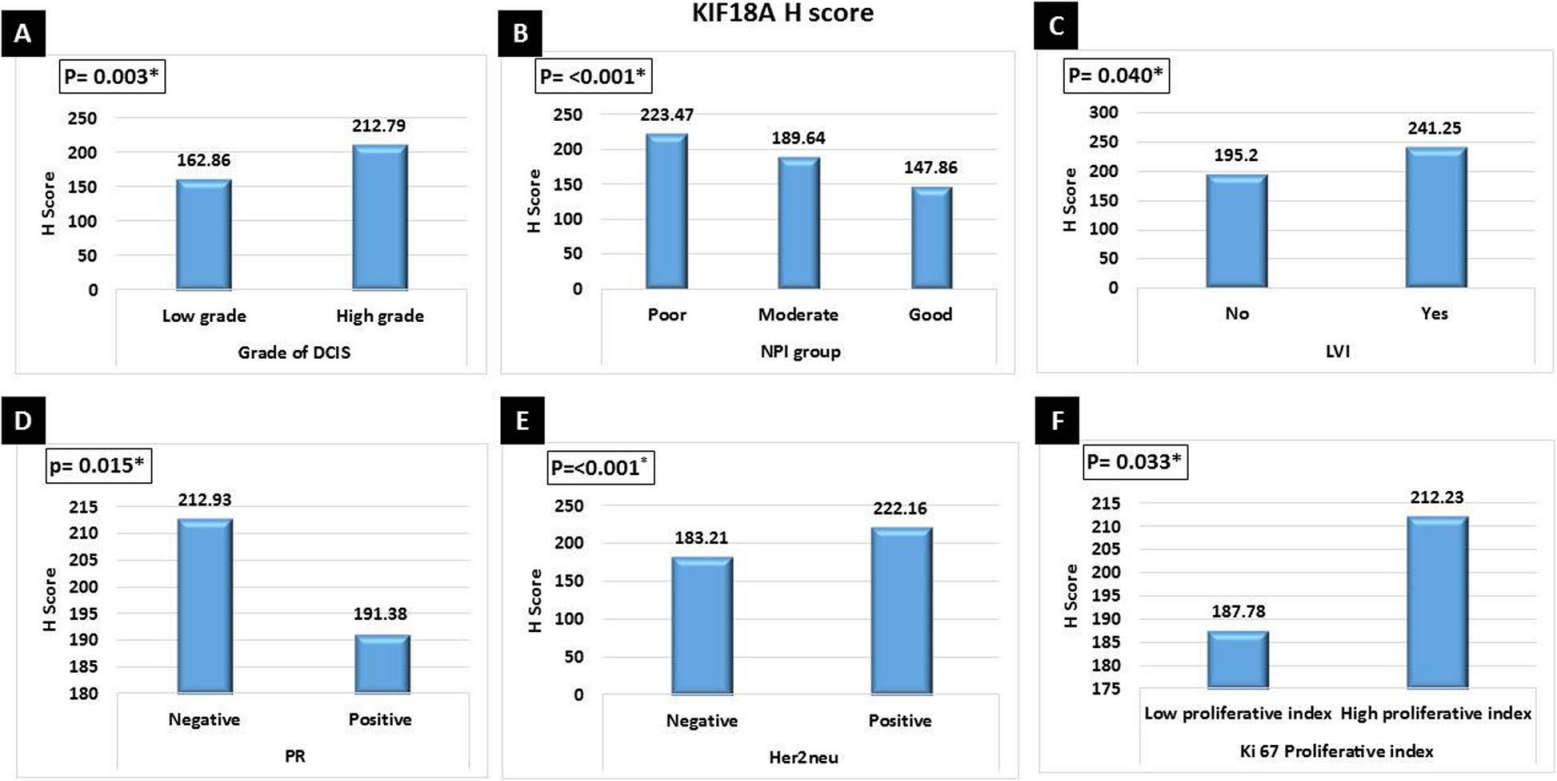
Significant relation between high KIF18A *H* score value expression and **A** high grade of associated DCIS (*p*=0.003), *B* poor NPI (*p*=0.001), *C* presence of lymphovascular invasion (*p*=0.040), *D* PR negative (*p*=0.015), *E* Her2neu positive (*p*<0.001), and *F* high Ki67 proliferative index (*p*=0.033)

**Fig. 4:**
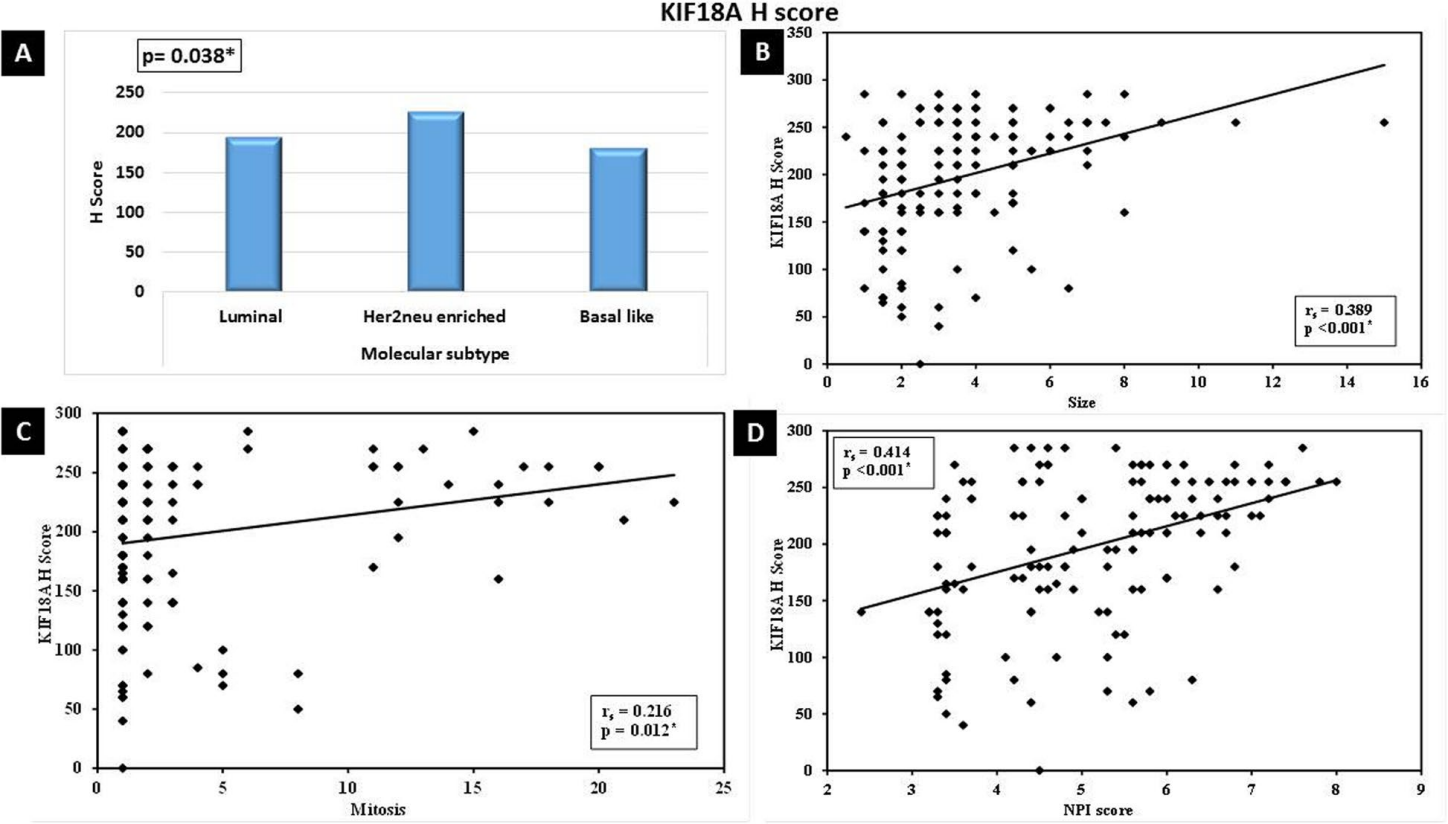
Significant relation between high KIF18A H Score value expression and **A** Her2neu-enriched molecular subtype breast cancer (*p*=0.038). Significant direct correlation between high KIF18A *H* score value and **B** large tumor size (*p*<0.001), **C** high mitosis (*p*< 0.012), and **D** high NPI score (*p*<0.001)

There was a significant association between high KIF18A *H* score expression and high tumor grade (*p*<0.001), advanced tumor stage grouping (*p*<0.009), nodal metastasis (*p*=0.001), advanced AJCC stage grouping (*p*<0.002), distant metastasis (*p*=0.015) (Fig. 2), associated high-grade DCIS (*p*<0.003), poor NPI (*p*<0.001), presence of lympho-vascular invasion (*p*<0.040), PR negative (*p*<0.015), Her2neu positive (*p*<0.001), high Ki67 proliferative index (*p*<0.033) (Fig. 3), and Her2neu enriched breast cancer (*p*<0.038) (Fig. 4).

Furthermore, there was a significant direct correlation between high KIF18A *H* score value and large tumor size (*p*<0.001), high mitosis (*p*<0.012), and high NPI score (*p*<0.001) (Fig. 4).

### Expression of β-catenin in studied groups (IDC and control normal breast tissue groups)

**Fig. 5:**
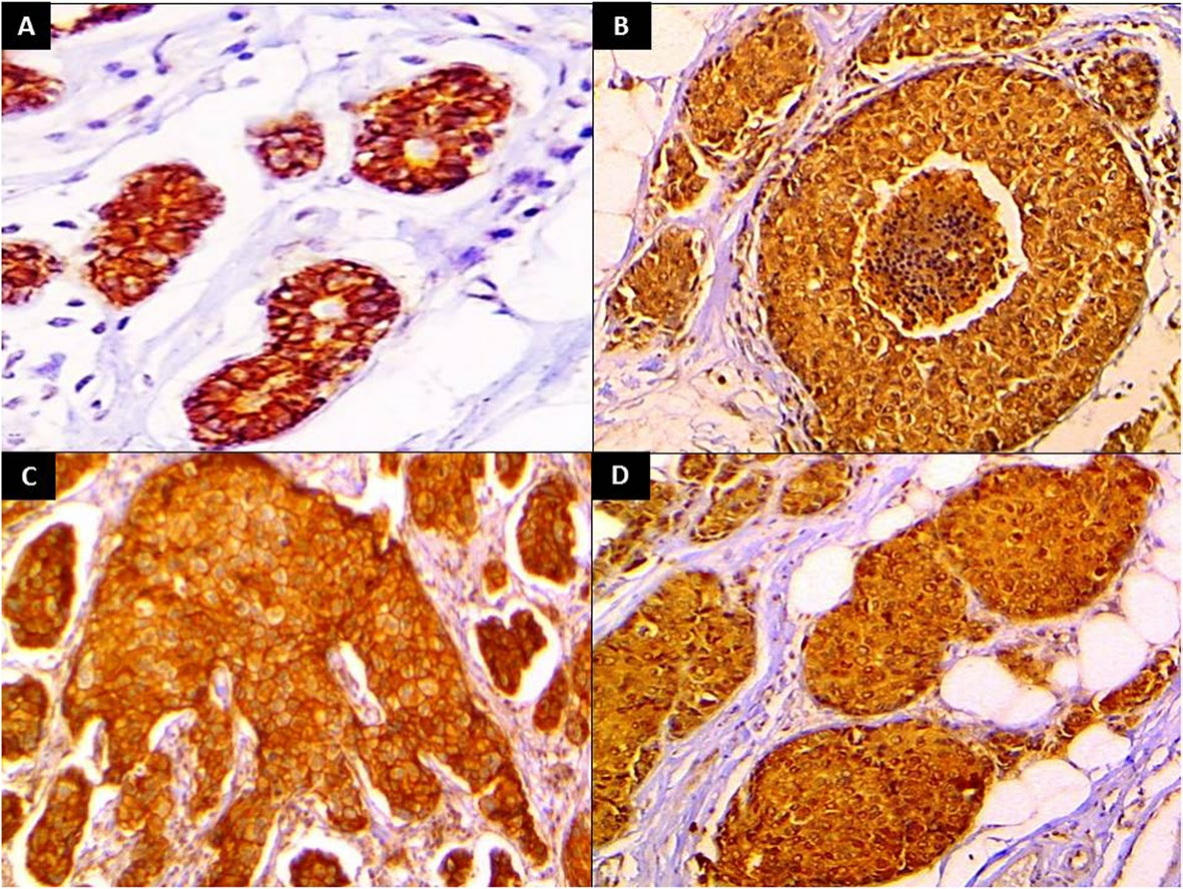
β-catenin immunostaining showed **A** positive membranous expression in normal breast tissue (IHC x100). Infiltrating duct carcinoma showing **B** strong cytoplasmic expression of β-catenin (IHC x400), **C** moderate membranous± cytoplasmic expression of β-catenin (IHC x400), and **D** strong nucleocytoplasmic expression of β-catenin (IHC x400)

All control group specimens showed positive membranous expression of β-catenin immunostaining (100%). β-catenin *H* score ranged from 210 to 285 with a mean ±SD of 250.13±25.05 and a median of 255 (Fig. 5).

Ninety-two IDC cases (68.1%) showed membranous± cytoplasmic pattern of β-catenin expression while 43 cases (31.9%) showed cytoplasmic± nuclear pattern of expression. β-catenin *H* score ranged from 50 to 285 with a mean± SD of 197.59±63.75 and a median of 210 (Fig. 5).

### Comparison between IDC and control normal breast tissue groups as regards of β-catenin expression

There was a highly significant difference between IDC and control normal breast tissue groups regarding β-catenin aberrant expression (nucleo-cytoplasmic or cytoplasmic staining), percent, intensity, and mean *H* score expression (*p*<0.001 for all) (Table 3).

**Table 3:** Comparison between the studied groups (IDC and control normal breast tissue groups) regarding β-catenin immunostaining

β-catenin		IDC cases (*n* = 135)		Control normal breast (*n* = 40)		Test of sig.	*p*
		No.	%	No.	%		
Positivity	Membranous	0	0	40	100.0		
	Cytoplasmic	92	68.1	0	0	*χ*^2^=175.0^*^	<0.001^*^
	Nucleocytoplasmic	43	31.9	0	0.0		
Percent	Min.–Max.	45.0–95.0		70.0–95.0		*U*=1688.0^*^	<0.001^*^
	Mean ± SD.	75.74 ± 11.68		83.37 ± 8.35			
	Median (IQR)	80.0 (70.0–85.0)		85.0 (75.0–90.0)			
Intensity	Mild	13	9.6	0	0.0	*χ*^2^=17.949^*^	<0.001^*^
	Moderate	32	23.7	0	0.0		
	Strong	90	66.7	40	100.0		
	Mild + moderate	45	33.3	0	0.0	*χ*^2^=17.949^*^	<0.001^*^
	Strong	90	66.7	40	100.0		
*H* score	Min.–Max.	50.0–285.0		210.0–285.0		*U*=1322.0^*^	<0.001^*^
	Mean ± SD.	197.59 ± 63.75		250.13 ± 25.05			
	Median (IQR)	210.0 (157.5–247.5)		255.0 (225.0–270.0)			
	Low (<210)	55	40.7	0	0.0	*χ*^2^=23.765^*^	<0.001^*^
	High (>210)	80	59.3	40	100.0		

*IQR* interquartile range, *SD* standard deviation, *U* Mann-Whitney test, *χ*^*2*^ chi-square test, *FE* Fisher’s exact, *p p* value for comparing between the studied groups

^*^Statistically significant at *p* ≤ 0.05

#### Relationship between β-catenin expression and clinicopathological data in IDC cases (*n* = 135)

**Fig. 6:**
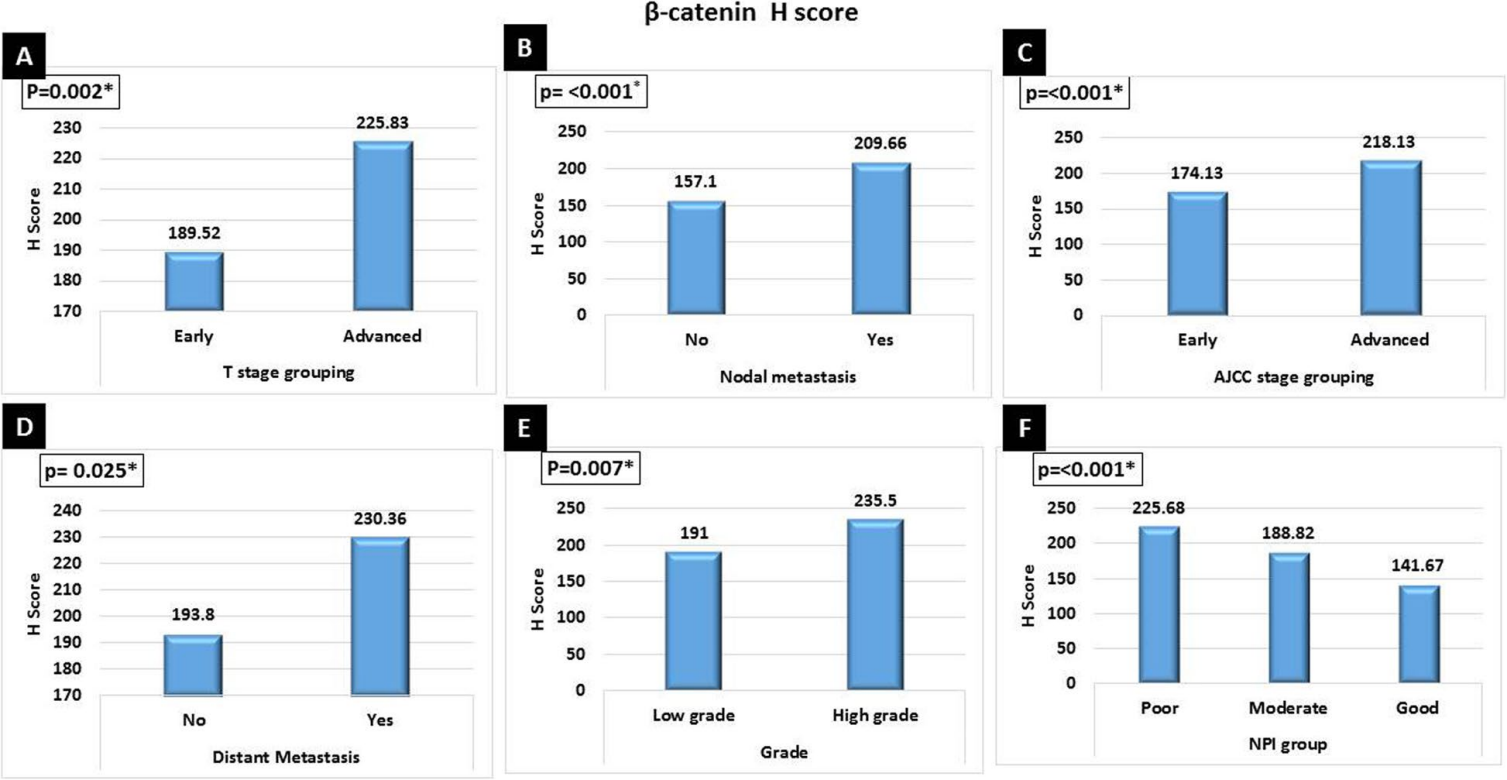
High β-catenin *H* score was significantly in favor of **A** advanced tumor stage grouping (*p*=0.002), **B** nodal metastasis (*p*<0.001), **C** advanced AJCC stage grouping (*p*<0.001), **D** presence of metastasis (*p*=0.025), **E** high tumor grade (*p*=0.007), and **F** poor NPI group (*p*<0.001)

**Fig. 7:**
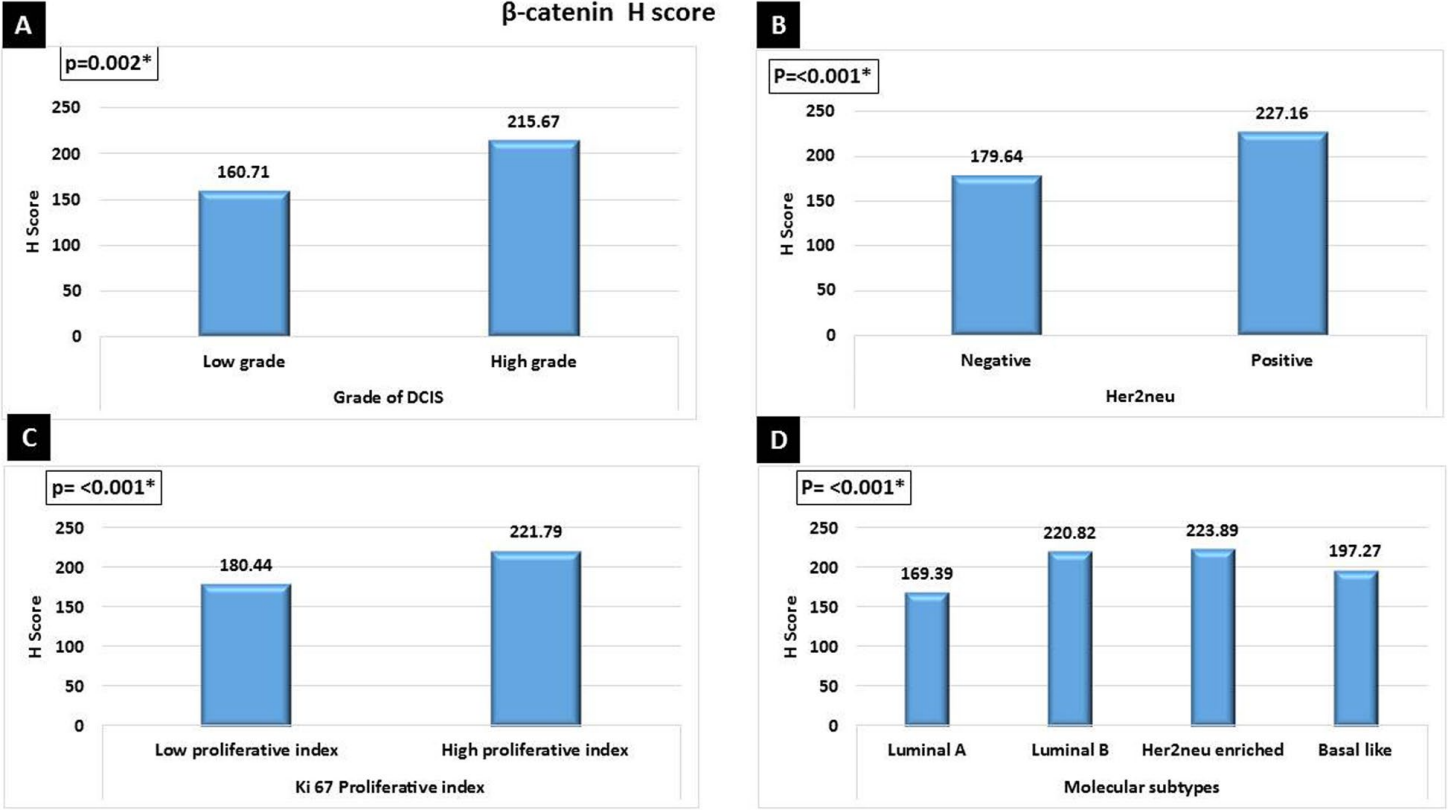
High β-catenin *H* score was significantly in favor of **A** high grade of associated DCIS (*p*=0.002), **B** Her2neu positive (*p*<0.001), **C** high Ki67 proliferative index (*p*<0.001), and **D** Her2neu-enriched breast cancer (*p*<0.001)

High β-catenin *H* score was significantly in favor of advanced tumor stage grouping (*p*<0.002), nodal involvement (*p*<0.001), advanced AJCC stage grouping (*p*<0.001), presence of metastasis (*p*<0.025), high tumor grade (*p*<0.007), poor NPI group (*p*<0.001), associated high-grade DCIS (*p*<0.002), Her2neu positive (*p*<0.001), high Ki67 proliferative index (*p*<0.001), and Her2neu enriched breast cancer (*p*<0.001) (Figs. 6 and 7). There was no significant difference between nucleo-cytoplasmic or cytoplasmic aberrant β-catenin expression as regards clinicopathological parameters of breast carcinoma cases.

### Relationship between KIF18A and β-catenin expression in IDC cases

**Fig. 8:**
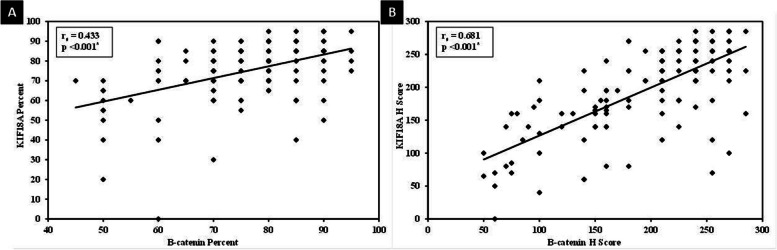
Highly significant direct correlation between KIF18A and β-catenin regarding **A** percent and **B** *H* score expression (*r*_*s*_ =0.433 and 0.681, respectively, *p*<0.001 for both)

There was a highly significant relationship between the intensity of KIF18A and β-catenin expression in the breast carcinoma group, as most of the cases that showed strong KIF18A expression also showed strong β-catenin (*p*<0.001). In addition, there was a highly significant direct correlation between KIF18A and β-catenin expression regarding percent (*r*_*s*_ =0.433, *p*<0.001) and *H* score values (*r*_*s*_ =0.681, *p*<0.001) (Fig. 8).

### Survival

Overall survival data was available for 102 cases out of the studied cases (75.56%). The follow-up period extended from January 2018 to December 2022. The survival time ranged from 9 to 52 months with a mean± SD of 28.24±13.52 months and a median of 30 months. Twenty-eight patients died of their disease through the period of follow-up (20.74%).

#### Univariate overall survival analysis of the studied clinicopathological parameters (*n*= 102)

**Fig. 9:**
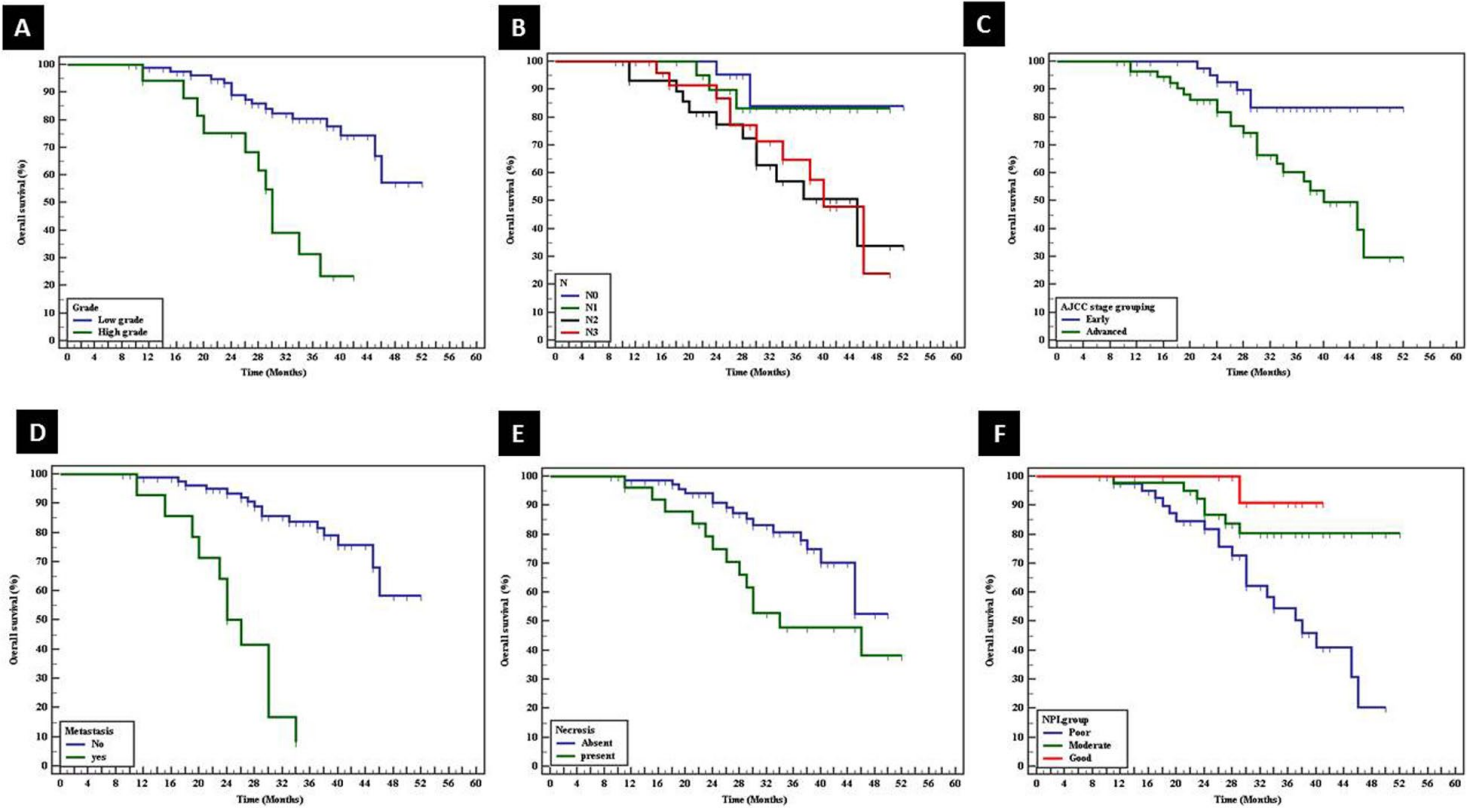
Univariate analysis of studied cases revealed the good prognostic impact of **A** low-grade tumors (*p*<0.001), **B** N0 nodal stage (*p*<0.033), **c** early AJCC staging group (*p*<0.003), **D** absence of metastasis (*p*˂0.001), **E** absence of necrosis (*p*<0.026), and **F** good NPI (*p*<0.002)

**Fig. 10:**
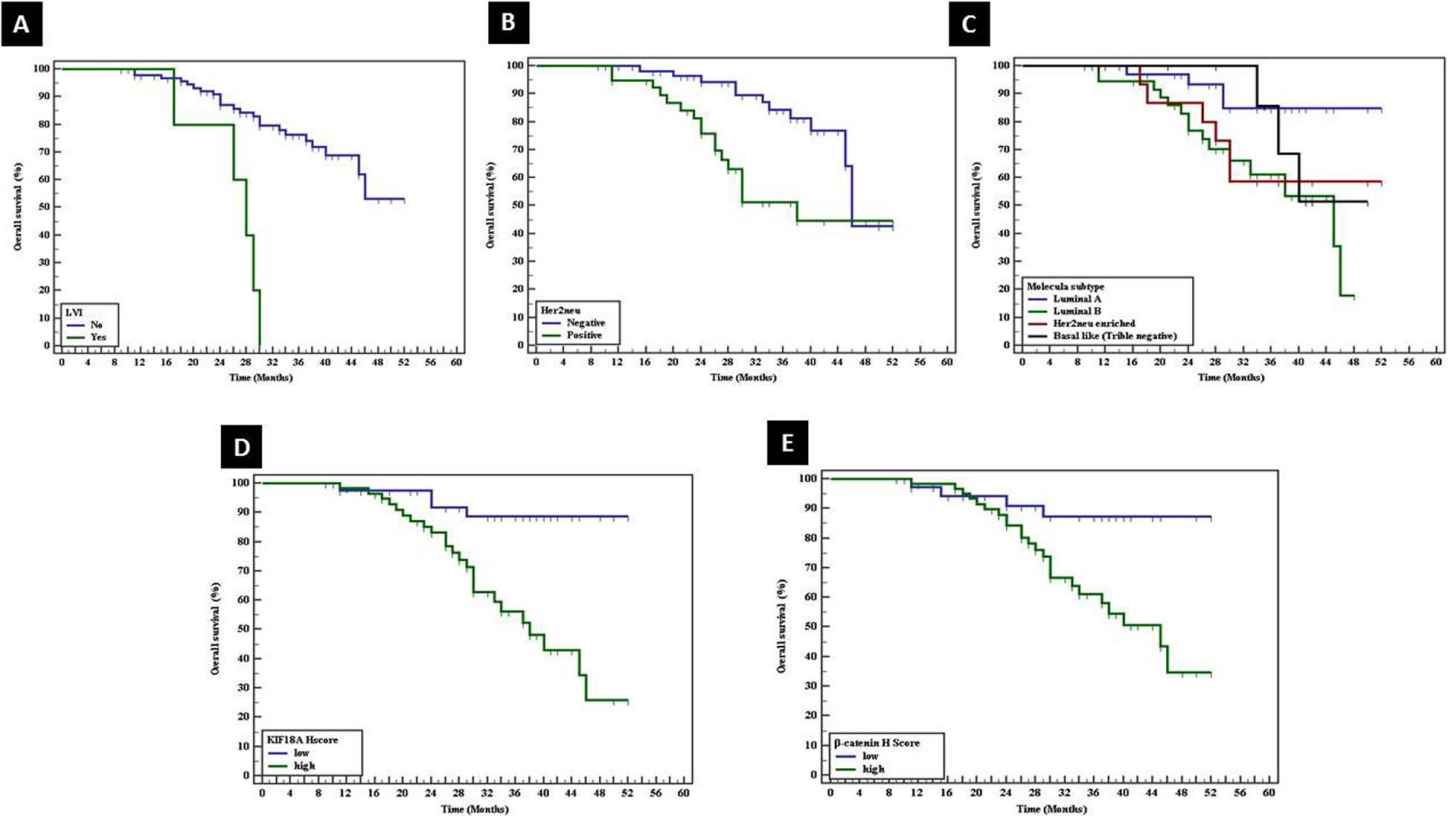
Univariate analysis of studied cases revealed the good prognostic impact of **A** absence of lympho-vascular invasion (*p*˂0.001), **B** negative Her 2neu (*p*<0.003), **C** luminal A molecular subtype (*p*<0.044), **D** low KIF18A *H* score (*p*<0.001), and **E** low β-catenin *H* score (*p*<0.006)

Univariate analysis of studied cases revealed the good prognostic impact of low-grade tumors (*p*<0.001), N0 nodal stage (*p*<0.033), early AJCC staging group (*p*<0.003), absence of metastasis (*p*˂0.001), absence of necrosis (*p*<0.026), good NPI (*p*<0.002), absence of lympho-vascular invasion (*p*˂0.001), negative Her 2neu (*p*<0.003), and luminal A molecular subtype (*p*<0.044) (Table 4) (Figs. 9 and 10).

**Table 4:** Univariate overall survival of cases as regards clinicopathological parameters

**Variable**	**Overall survival (months)**			
	**Mean survival time**	**SE**	**Log rank**	***P* value**
**T stage grouping**				
Early	43.636	1.710	2.102	0.147
Advanced	38.981	2.791		
***N***				
N0	48.090	2.076	8.747	0.033^*^
N1	45.651	2.304		
N2	37.698	2.926		
N3	38.587	2.536		
**AJCC stage grouping**				
Early	47.693	1.616	8.589	0.003^*^
Advanced	38.315	2.035		
**Metastasis**				
No	45.365	1.426	42.814	<0.001^*^
Yes	25.048	1.901		
**Grade**				
Grade I	42.0	0.0	17.218	<0.001^*^
Grade II	38.277	0.908		
Grade III	29.712	2.284		
Low grade	44.680	1.510	17.0	<0.001^*^
High grade	29.947	2.367		
**Necrosis**				
Absent	43.048	1.541	4.937	0.026^*^
Present	37.020	2.954		
**NPI group**				
Poor	36.187	2.040	12.949	0.002^*^
Moderate	46.349	1.936		
Good	39.909	1.040		
**Lymphovascular invasion**				
No	43.415	1.489	18.531	<0.001^*^
Yes	26.000	2.345		
**ER**				
Negative	42.055	2.620	0.106	0.745
Positive	42.331	1.803		
**PR**				
Negative	40.609	2.398	1.329	0.249
Positive	43.274	1.868		
**Her2neu**			8.626	0.003^*^
Negative	44.946	1.758		
Positive	37.223	2.574		
**Ki 67 Proliferative index**				
Low proliferative index	44.750	1.896	2.519	0.112
High proliferative index	39.817	2.140		
**Molecular subtype**				
Luminal	106	78.5	8.075	0.044^*^
Her2neu-enriched	40.840	3.599		
Basal-like (triple negative)	43.771	2.659		

^*^significant *p* value

#### Univariate overall survival analysis of the studied immunohistochemical markers (KIF18A and β-catenin) (*n*= 102)

Prolonged OS was significantly associated with mild intensity of KIF18A (*p*<0.003) and low KIF18A *H* score (*p*<0.001). Similarly, prolonged OS was significantly correlated with mild and moderate intensity of β-catenin (*p*<0.042) and low β-catenin *H* score (*p*<0.006) (Table 5).

**Table 5:** Univariate overall survival analysis of cases as regards KIF18A and β-catenin expression

	**Variable**	**Overall survival (months)**			
		**Mean survival time**	**SE**	**Log rank**	***P* value**
**KIF18A**	**Positivity**				
	Negative			–	–
	Positive	42.269	1.485		
	**Localization everywhere**				
	Cytoplasmic	43.694	2.224	0.429	0.513
	Nucleocytoplasmic	41.345	1.948		
	Nuclear	–	–		
	**Intensity**				
	Mild	44.0	0.0	11.443	0.003^*^
	Moderate	39.961	1.452		
	Strong	38.806	1.820		
	Mild + moderate	49.707	1.595	11.300	0.001^*^
	Strong	38.806	1.820		
	***H* score**				
	Low (<210)	48.666	1.592	13.613	<0.001^*^
	High (>210)	37.478	1.942		
**β-catenin**	**Membranous**				
	No	41.805	1.740	0.169	0.681
	Yes	42.873	2.419		
	**Positivity**				
	Cytoplasmic	44.007	1.692	3.373	0.066
	Nucleocytoplasmic	38.307	2.841		
	Membranous	–	–		
	**Intensity**				
	Mild	44.0	0.0	5.168	0.075
	Moderate	39.961	1.452		
	Strong	38.806	1.820		
	Mild + moderate	47.061	2.305	4.141	0.042^*^
	Strong	40.366	1.754		
	***H* score**				
	Low (<210)	47.988	1.897	7.533	0.006^*^
	High (>210)	39.323	1.838		

^*^significant *p* value

### Multivariate Cox regression analysis for detection of independent parameters in IDC cases

Multivariate Cox regression analysis showed that the molecular subtype of the tumor (*p*<0.006) and distant metastasis (*p*<0.018) were the most independent prognostic factors affecting overall survival in the studied IDC cases (Table 6).

**Table 6:** Multivariate Cox regression analysis of overall survival (months) for IDC cases

	**B**	**SE**	**HR**	**95% CI**		**Sig.**
				**LL**	**UL**	
**Grade**	0.340	0.599	1.404	0.434	4.542	0.571
**Nodal stage**	−0.654	0.507	0.520	0.192	1.404	0.197
**AJCC stage grouping**	2.441	1.369	11.485	0.785	168.051	0.075
**Metastasis**	1.401	0.591	4.060	1.274	12.936	0.018^*^
**Necrosis**	0.224	0.581	1.251	0.400	3.908	0.700
**NPI group**	0.430	1.106	1.537	0.176	13.435	0.698
**Lymphovascular invasion**	1.130	0.783	3.097	0.668	14.367	0.149
**Molecular subtypes**	−1.635	0.596	0.195	0.061	0.627	0.006*
**Her2neu**	0.531	0.527	1.700	0.606	4.775	0.313
**KIF18A intensity**	0.517	1.270	1.677	0.139	20.211	0.684
**KIF18A H score**	1.379	1.086	3.971	0.472	33.399	0.204
**β-catenin intensity**	−11.010	106.447	0.0	0.000	–	0.918
**β-catenin *H* score**	9.960	106.445	21,169.442	0.000	–	0.925

*HR* hazard ratio, *C.I* confidence interval, *LL* lower limit, *UL* upper limit

^#^All variables with *p*<0.05 were included in the multivariate

^*^Statistically significant at *p* ≤ 0.05

## Discussion

Breast cancer is currently the second most common cause of Egyptian cancer mortality after hepatocellular carcinoma. Recent data analysis stated that breast cancer in Egyptian patients is diagnosed at a younger age, advanced stage, and comprises more aggressive subtypes than in other developed countries [24]. Therefore, there is a need to discover biomarkers that take part in tumor pathogenesis and metastasis and help to predict prognosis and new targeted therapies.

KIF18A is a member of the kinesin-8 family that is tumor-related by regulating microtubule dynamics and mitosis. Dysfunction of KIF18A affects chromosome instability and promotes carcinogenesis [25]. β-catenin is an oncogene regulating cell–cell adhesion and has a critical role in the Wnt signaling pathway and carcinogenesis [26]. To the best of our knowledge, the relation between KIF18A and β-catenin in breast cancer was not previously investigated.

In this study, IDC cases showed significantly higher KIF18A percent, intensity, and mean *H* score values than control normal breast tissue. Two previous studies reported similar results [8, 9], and this suggests that KIF18A is involved in breast carcinogenesis.

High KIF18A *H* score values were associated with poor prognostic factors such as high grade, advanced stage, nodal metastasis, distant metastasis, high grade of associated DCIS, poor NPI, presence of lympho-vascular invasion, PR negative, high Ki67 status, and Her2neu-enriched breast cancer.

Furthermore, high KIF18A H score values showed a significant direct correlation with large tumor size, high mitosis, and high NPI score. This poor prognostic significance of KIF18A was documented by Zhang et al. 2010 and Kasahara et al. (2016). They demonstrated a significant association between KIF18A expression and lymph node metastasis, tumor size, lymphatic invasion, and tumor recurrence [8, 9]. Kasahara et al. (2016) concluded that in biopsies that showed low KIF18A expression before surgery, doctors can avoid sentinel node biopsy in selected patients with normal axilla [9]. Furthermore, Alfarsi et al. 2019 found that high KIF18A mRNA was significantly associated with poor NPI, higher tumor grade, and larger tumor size in ER + breast cancer. Therefore, they suggested that KIF18A can predict poor benefits from endocrine treatment for these patients [10]. Taken together, KIF18A expression in breast cancer could be used to tailor treatment options for breast cancer patients.

In this study, all control group specimens showed membranous expression of β-catenin immunostaining (100%), while malignant cases showed an aberrant pattern of its expression in the form of membranous± cytoplasmic in 68.1% and cytoplasmic± nuclear pattern of expression in 31.9%. There was a highly significant difference between IDC and control normal breast tissue groups as regards β-catenin aberrant expression, percent, intensity, and mean *H* score values.

Varma et al. (2020) reported that abnormal β-catenin expression was seen in 80% of IDC cases and may act as an oncogene [26]. Wang et al. (2015) demonstrated that 58% of breast cancer patients showed abnormal β-catenin expression [27]. It was found that aberrant Wnt signaling or mutation of the β-catenin gene leads to nuclear accumulation of β-catenin and breast cancer development. Also, cytoplasmic expression of β-catenin could indicate malignant transformation in breast tissue [28]. So, aberrant β-catenin expression may act as a proto-oncogene, involved in breast cancer pathogenesis.

In the current work, high β-catenin H score in IDC cases was significantly associated with advanced T stage, nodal metastasis, advanced AJCC stage, higher tumor grade, presence of necrosis, increased mitosis, poor NPI group, high grade of associated DCIS, ER negative, PR negative, Her2neu positive, high Ki67 proliferative index, Her2neu-enriched breast cancer, and non-luminal molecular subtype. Similarly, Wang et al. 2015 found that β-catenin was significantly correlated with a Ki-67 labeling index (>14%) and high tumor grade [27]. Moreover, other studies confirmed the association of β-catenin with poor prognostic parameters as increased invasion and metastasis [26, 29]. Taken together, these results may confirm that β-catenin may promote breast cancer cell proliferation and could be used as a prognostic marker of advanced breast cancer.

However, other conflicting and contradictory results were reported by Nakopoulou et al. (2006), as they found that cytoplasmic phospho-β-catenin was associated with a favorable tumor phenotype including small tumor size, early stage, and low Ki-67 status. They explained that phospho-*β*-catenin may affect tumors’ phenotype and prognostic value, according to its subcellular distribution either cytoplasmic or nuclear [30].

To the best of our knowledge, the relationship between KIF18A and β-catenin in breast cancer was not previously investigated. Only another protein of the kinesin superfamily (KIF18B) was correlated with β-catenin in cervical cancer [12] and was suggested to act in breast cancer by activating the Wnt/β-catenin signaling pathway [31]. In this study, there was a highly significant relationship between the intensity of KIF18A and β-catenin expression in IDC. In addition, a highly significant direct correlation between KIF18A and β-catenin percent and *H* score expression was demonstrated. Therefore, KIF18A may be involved in breast carcinogenesis by activating β-catenin.

Univariate analysis of studied IDC cases revealed that prolonged overall survival was in favor of low-grade tumor, N0 nodal stage, early AJCC staging, absence of metastasis, absence of necrosis, and absence of lympho-vascular invasion. In addition, multivariate Cox regression analysis showed that the molecular subtype of the tumor and distant metastasis were the most independent prognostic factors affecting overall survival. These prognostic factors that affect the survival of breast cancer patients were demonstrated by other previous studies [32–34].

In this work, a high KIF18A H score was associated with shorter overall survival. Similar results were reported by previous studies that showed KIF18A as an independent predictive factor for the lymph node metastasis and disease-free survival [9, 10]. These results supported that KIF18A is associated with aggressive tumors.

In this work, a high β-catenin *H* score was associated with shorter overall survival. Similarly, Lin et al. 2000 reported that activated β-catenin was associated with shorter overall survival. In addition, they considered β-catenin as a strong independent prognostic factor for breast cancer survival [35]. There was no significant difference between nucleo-cytoplasmic or cytoplasmic aberrant β-catenin expressions as regards overall survival of breast carcinoma cases.

Nakopoulou et al. (2006) reported that nuclear β-catenin expression correlated with reduced overall survival, while cytoplasmic β-catenin expression was associated with longer overall survival [30]. These results supported the poor prognostic significance of aberrant β-catenin expression and confirmed its association with aggressive tumors.

In conclusion, KIF18A could be involved in breast carcinogenesis by activating β-catenin. Overexpression of KIF18A and aberrant expression of β-catenin are considered proto-oncogenes of breast cancer development. KIF18A and β-catenin could be poor prognostic markers and predictors of aggressive behavior of breast cancer.

### Limitations of the study

The limited number of cases and short follow-up period besides the absence of data considering response to therapy limits this study.

## Data Availability

The datasets supporting the conclusions of this article are included within the article.

## Abbreviations

IDC: Infiltrating duct carcinoma
AJCC: American Joint Committee on Cancer
NPI: Nottingham prognostic index
TILs: Tumor-infiltrating lymphocytes
DCIS: Ductal carcinoma in situ
TMA: Tissue microarray
OS: Overall survival
